# Effect of disclosure of HIV status on patient representation and adherence to clinic visits in eastern Uganda: A propensity-score matched analysis

**DOI:** 10.1371/journal.pone.0258745

**Published:** 2021-10-19

**Authors:** Jonathan Izudi, Stephen Okoboi, Paul Lwevola, Damazo Kadengye, Francis Bajunirwe

**Affiliations:** 1 Department of Community Health, Faculty of Medicine, Mbarara University of Science and Technology, Mbarara, Uganda; 2 Institute of Public Health and Management, Clarke International University, Kampala, Uganda; 3 Infectious Diseases Institute, School of Medicine, Makerere University College of Health Sciences, Kampala, Uganda; 4 African Population and Health Research Center (APHRC), APHRC Campus, Nairobi, Kenya; Fred Hutchinson Cancer Research Center, UNITED STATES

## Abstract

**Background:**

Disclosure of human immunodeficiency virus (HIV) status improves adherence to antiretroviral therapy (ART) and increases the chance of virological suppression and retention in care. However, information on the effect of disclosure of HIV status on adherence to clinic visits and patient representation is limited. We evaluated the effects of disclosure of HIV status on adherence to clinic visits and patient representation among people living with HIV in eastern Uganda.

**Methods:**

In this quasi-randomized study, we performed a propensity-score-matched analysis on observational data collected between October 2018 and September 2019 from a large ART clinic in eastern Uganda. We matched participants with disclosed HIV status to those with undisclosed HIV status based on similar propensity scores in a 1:1 ratio using the nearest neighbor caliper matching technique. The primary outcomes were patient representation (the tendency for patients to have other people pick-up their medications) and adherence to clinic visits. We fitted a logistic regression to estimate the effects of disclosure of HIV status, reported using the odds ratio (OR) and 95% confidence interval (CI).

**Results:**

Of 957 participants, 500 were matched. In propensity-score matched analysis, disclosure of HIV status significantly impacts adherence to clinic visits (OR = 1.63; 95% CI, 1.13–2.36) and reduced patient representation (OR = O.49; 95% CI, 0.32–0.76). Sensitivity analysis showed robustness to unmeasured confounders (Gamma value = 2.2, *p* = 0.04).

**Conclusions:**

Disclosure of HIV status is associated with increased adherence to clinic visits and lower representation to collect medicines at the clinic. Disclosure of HIV status should be encouraged to enhance continuity of care among people living with HIV.

## Introduction

Globally, Human Immunodeficiency Virus (HIV) remains a major public health problem and has so far claimed 35 million lives. At the end of 2019, approximately 38 million people were living with HIV and nearly 1.7 million become newly infected with HIV [1]. Of all the regions in the world, the African region is the most affected by HIV, with at least 25 million people living with HIV besides being home to almost two-thirds of the global new HIV infections [2]. In Uganda, estimates indicated that 1.4 million people were living with HIV at the end of 2019, and of this, 88% knew their HIV status while 87% of those who knew their HIV status are on HIV treatment [3]. Data about disclosure of HIV status at the national level is lacking but observational studies conducted in recent years and different populations indicate that at least 80% of people living with HIV have disclosed their HIV status to someone [4–6].

Disclosure of HIV status involves revealing one’s HIV positive status to a sexual partner(s), family members, or others in their social circle [7], and is considered a key component of the positive health, dignity, and prevention (PHDP) package within Uganda’s HIV programming. The package offers an option for a provider and/or counselor-mediated or supported disclosure for people who are having difficulty disclosing their HIV status [3, 8]. The disclosure of HIV status has several benefits namely, improved adherence to medications, access to essential services, reduced psychological distress, increased likelihood of appropriate disclosure to other people, better engagement in HIV-related care, improved understanding of HIV and related conditions, and enhanced uptake of the PHDP package [8], improved quality of life, better immune recovery as reflected by rising CD4 cell counts, and viral load suppression [9, 10]. Also, disclosure of HIV status is associated with a higher likelihood of retention [11] while non-disclosure of HIV status is associated with an increased risk of loss to follow-up [12].

Furthermore, disclosure of HIV status is associated with a higher likelihood of condom use, an increased social support, and knowledge of the partner’s HIV status [7].

Adherence to HIV clinic visits has several benefits such as a lower risk of mortality [13], adherence to medication, slower progression of disease, higher odds of viral load suppression and immune recovery [14, 15], and lower risk of hospitalization [16]. In situations where a patient is not able to attend a clinic visit in person, they may send a representative to collect HIV medications for them, and this patient representation is an allowable practice in Uganda. Patient representation is an alternative to individual clinic visits and a recognized form of clinic attendance. Although acceptable, patient representation may result in loss of benefits of individual clinic attendance namely, the provision of ongoing counseling and support, and missed clinical, immunologic, and virologic monitoring which are intended to monitor treatment success. Numerous benefits of disclosure of HIV status have been described, but there are limited studies on the effect of disclosure of HIV status on patient representation and adherence to clinic visits. We hypothesized that disclosure of HIV status is associated with lower patient representation and higher adherence to clinic visits [17]. Therefore, the primary objective of this study was to assess the effect of disclosure of HIV status on patient representation and adherence to clinic visits among people living with HIV in an ART clinic in eastern Uganda.

## Methods and materials

### Data source and study setting

The data (S1 File) for this study were drawn from the routine health care records of the HIV clinic at Kidera Health Center HC IV, the largest ART clinic in Buyende district in rural eastern Uganda.

The population of the district is 323, 067 people of which 50.9% (164,452) are females [18]. The health facility serves as the referral site for HIV care in the district, serving about 32% of people living with HIV in the district. The health facility has a catchment population of approximately 60,000 people. Besides providing comprehensive HIV care, the health facility provides promotion, preventive, curative, and rehabilitative health services to the catchment population.

## Study population

The study population consisted of a census of people living with HIV started on ART between October 2018 and September 2019. The eligible participants included those aged ≥15 years and enrolled in care for ≥6 months during the review period. We excluded participants transferred to other health facilities because it was logistically infeasible to follow all of them and obtain data about their HIV disclosure status. We also excluded participants who were documented dead to prevent a biased estimate of the HIV disclosure effect. Further, we excluded participants whose disclosure of HIV status had occurred after the study outcomes as this would result into an inaccurate measure of the temporal relationship between disclosure of HIV status and the study outcomes.

### The operation of the ART clinic

The ART clinic is run by a clinical officer, two nurses, a counselor, and two volunteers who provide clinical care, nursing care, psychosocial counseling support, and health education to people living with HIV. The clinic runs twice a week and has special clinics for children and adolescents.

Patients who are stable on ART namely those, 1) who have been on their current ART regimen for more than a year; 2) with undetectable viral load at the most recent test defined as viral load less than 1000 copies per ml in the last 12 months; 3) in the World Health Organization (WHO) clinical stages I and II; and, 4) who have demonstrated good adherence defined as more than 95% ART adherence in the last six consecutive months [3], receive refills of anti-retroviral drugs (ARVs) to last 3–6 months. The rest of the patients are considered unstable and receive refills of anti-retroviral drugs (ARVs) to last one month.

To track adherence to clinic visits, the ART clinic maintains an appointment register where the scheduled clinic visit dates of all patients are captured. Although the study participants have different ART regimens, the clinic uses an appointment system that enables multi-month dispensing of drugs, usually is 1–2 months of refill. Adherence to the clinic visit is updated in real-time on clinic days by a records assistant. There is also a register for the missed appointment to record all patients who miss an appointment to enable follow-up either immediately through phone calls or home visits within 2–5 days.

To minimize non-adherence to clinic visits, reminders are sent before scheduled clinic visits, and for those who have failed to come to the ART clinic as scheduled, the ARV refills are done in the community. Once the ARV refills are completed, the relevant clinic registers are updated. For patients who fail to adhere to scheduled clinic visits, their HIV medications can be collected by a representative who in most cases is a treatment supporter. However, not more than 2 consecutive representations are allowed. Following the Uganda Ministry of Health HIV treatment guidelines [3], a patient is considered to have dropped from HIV care if he/she is lost for at least 3 months and 3 attempts to follow-up have been unsuccessful.

### Data abstraction

We abstracted data from the ART register for all eligible patients (patients started on ART between October 2018 and September 2019, aged ≥15 years, and enrolled in care for ≥6 months during the review period) using a standardized data abstraction tool. We received a waiver of informed consent from Clarke International University Research Ethics Committee (CIU-REC) to retrieve and analyze patient records since it would be impossible and logistically inefficient to reach all the patients (CIU-REC number CLARKE-2020-16).

### Study design

To measure the effects of an intervention, a randomized control trial (RCT) is the gold standard because randomization achieves comparability by balancing both measured and unmeasured participant characteristics across the intervention and treatment groups [19]. However, an RCT is not always feasible or ethical for interventions that are known to have certain positive benefits such as disclosure of HIV status. We, therefore, used observational data to approximate an RCT by creating two groups, distinguished by disclosure status (the exposure factor) but similar based on observed covariates, achieved using propensity-score matching [20]. Propensity-score matched analysis is a statistical approach that simulates an RCT by balancing all observed confounders or covariates across treatment groups except the treatment [21]. Since this analytic approach does not rely on true randomization, the study design is quasi-randomized [20].

### Measurements: Exposure and outcomes

The exposure in this study was the disclosure of HIV status measured as a dichotomous variable at the second visit (week 2) after initiation of ART and updated as treatment progresses.

Disclosure of HIV status was defined as revealing HIV positive status to a sexual partner(s), family members, or others in their social circle [7]. The exposed group consisted of participants with disclosed HIV status while the unexposed group comprised of participants with undisclosed HIV status. Our analysis considered the disclosure of HIV status that preceded the study outcomes to ensure a valid measure of effect. The study outcomes were patient representation and adherence to clinic visits in the past 6 months, measured following the Uganda Ministry of Health guidelines [22].

- **Patient representation:** This was measured as the practice where the individual patient does not show up at the HIV clinic on the scheduled date but delegates someone to pick up the medications for them. Participants who did not show up at the HIV clinic on one or more occasions in the past 6 months were considered to have been represented. This outcome was measure as a binary variable (yes or no).
- **Adherence to clinic visits**: This was a binary variable (yes or no) measured as adherence to the visit at the HIV clinic where the individual patient attends clinic on the date that he/she was scheduled or within seven days before or after the scheduled date. All participants who did not adhere to their scheduled visit within the recommended period (±7 days) were considered non-adherent to the clinic visits.
- **Matching covariates:** These included age in years (≤24, 25–50, and >50), sex (female or male), marital status (single, married, and separated), level of education (none, primary, secondary, tertiary, and above), availability of a source of income (no or yes), estimated distance from home to a health facility in kilometers (<5, 5–10, >10), ease of access to health facility (no or yes), current alcohol consumption (no or yes), current smoking status (no or yes), duration on ART in years (<5, 5–9, >10), receipt of pre-ART counseling (no or yes), receipt of pre-tuberculosis preventive therapy counseling (no or yes), and experience of tuberculosis preventive therapy-related side effects (no or yes).

### Statistical analysis

The analysis was performed in R statistical software and programming language [23], using the *MatchIt* [24] and *tableone* packages [25]. We performed a descriptive analysis to summarize categorical data like sex using frequencies and percentages and numerical data like age using the mean with standard deviations or median with interquartile range.

In the propensity score-matched analysis, we selected 12 matching covariates as already described. These covariates were selected because they are known to be associated with the study outcomes and exposure thus preserving the assumption of unconfoundedness of the association between the exposure and the outcome(s) [26, 27]. Under this assumption, treatment assignment and participant responses are conditionally independent after controlling for covariate effects that determine the assignment mechanism [28].

We generated propensity scores by regressing the matching covariates on disclosure of HIV status in a logit model and assessed the initial balance in propensity scores between the groups using a back-to-back histogram [29]. We then matched participants with undisclosed HIV status (the unexposed group) to participants with disclosed HIV status (the exposed group) on similar propensity scores [24]. We employed several matching approaches. Briefly, we used greedy matching approaches namely nearest neighbor matching with and without caliper adjustment [27]. Caliper is the distance within which the matches were considered. In nearest neighbor matching without caliper adjustment, one participant in the HIV disclosed group was selected at random and matched to one participant in the undisclosed HIV status with the closest propensity score. In nearest neighbor matching with caliper adjustment, the matching was performed within a caliper of 20% of the standard deviation of the propensity score to prevent bias from distant matches. The matching was performed without replacement.

We also employed optimal matching namely optimal pair matching and optimal full matching [28]. In optimal pair matching, we matched the participants in pairs and removed the unmatched pairs from the analysis. In optimal full matching, participants with disclosed HIV status were matched to those with undisclosed HIV status in the ratio of 1: many or many: 1. In addition, we performed exact matching where the participants were matched on the same value of propensity score [30]. We considered the best matching approach as one that balanced all the covariates between the disclosed HIV status and the undisclosed HIV status groups.

We assessed covariate balance using standardized mean differences (SMD) and considered an SMD less than 0.2 to confirm covariate balance [31]. We graphically explored covariate balance using a jitter plot and histogram, and we considered the distributional similarity of propensity scores as confirmatory of covariate balance [31, 32]. We saved the matched dataset for the outcome analysis.

In the unmatched and matched datasets, we separately performed outcome analysis with logistic regression fitted using the generalized linear model with a logit-link and binomial family and reported the results using the odds ratio (OR) and the corresponding 95% confidence interval (CI). The odds ratio indicates the measure of average treatment effect on the treated (ATT), a measure of the effect of HIV status disclosure for those with disclosed HIV status. We performed a sensitivity analysis using Rosenbaum Wilcoxon’s signed-rank test to check the robustness of our results to hidden bias [29] and assessed this by examining the p-values that there is no hidden bias with varying values of the sensitivity parameter gamma.

## Results

### Study profile and matching technique

**Fig 1:**
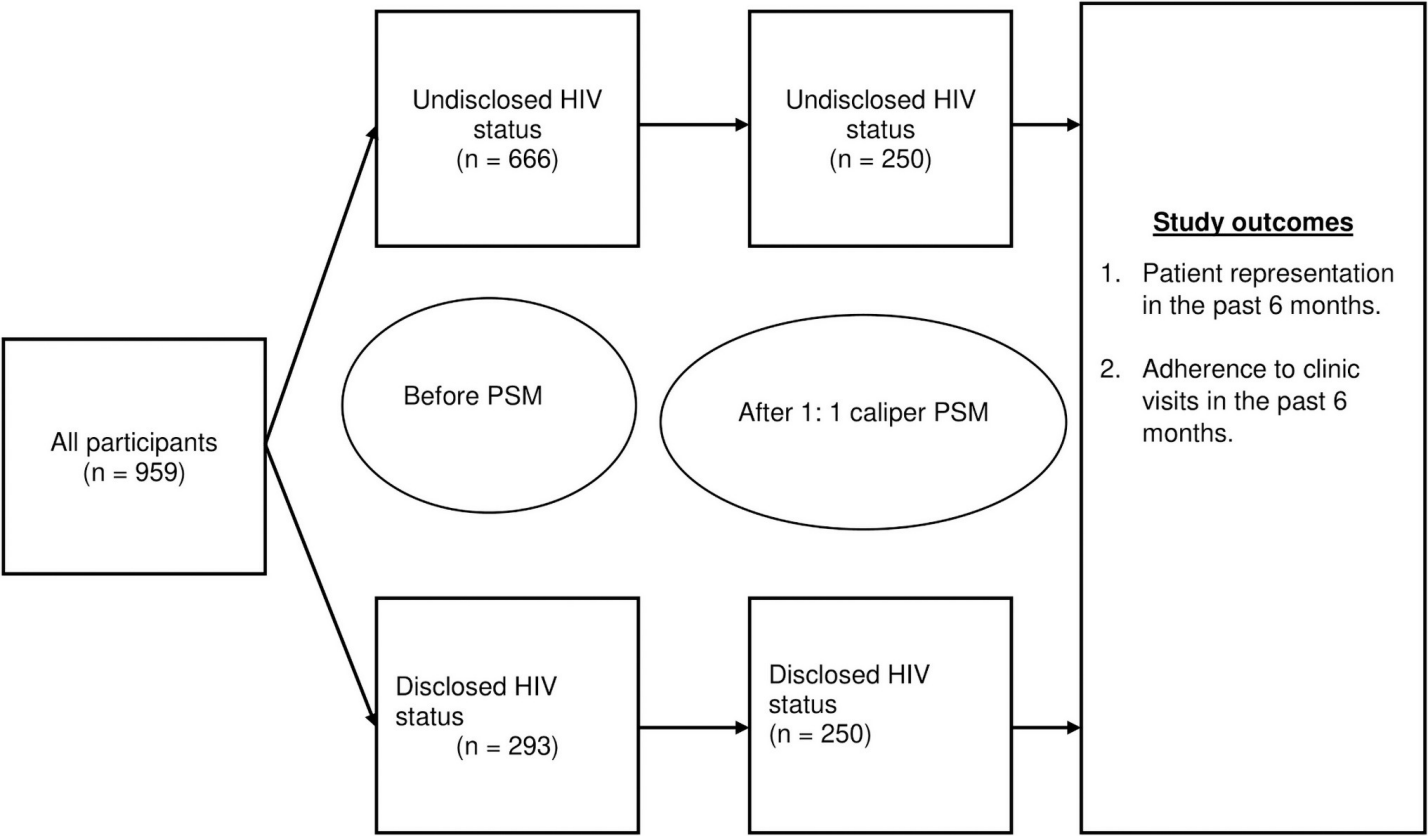
Study profile of HIV status disclosure before and after propensity score matching.

The dataset comprised 959 patients. Fig 1 summarizes our study profile and shows that of the 959 participants, 293 (30.6%) had disclosed HIV status in the unmatched data. We matched 500 participants in the ratio of 1:1 using the nearest neighbor caliper matching, representing 52.1% of the original data. The caliper used was 0.0265, which was the 20% of the standard deviation of the propensity score.

### Covariate balance before and after propensity-score matching

In the unmatched cohort data (Table 1), we observed systematic differences among participants with disclosed HIV status and undisclosed HIV status regarding the covariates of sex, marital status, and level of education, source of income, and distance and accessibility to a health facility.

**Table 1:** Distribution of participants’ characteristics before and after propensity-score matching.

		Original (Unmatched) cohort				PSM matched cohort			
Variable	Level		HIV status disclosure				HIV status disclosure		
	Sample size	All,	No,	Yes,	SMD	All,	No,	Yes,	SMD
		n = 959 (%)	n = 666 (%)	n = 293 (%)		n = 500 (%)	n = 250 (%)	n = 250 (%)	
Age categories (years)	≤24	104 (10.8)	73 (11.0)	31 (10.6)	0.023	58 (11.6)	30 (12.0)	28 (11.2)	0.059
	25–50	631 (65.8)	436 (65.5)	195 (66.6)		327 (65.4)	160 (64.0)	167 (66.8)	
	>50	224 (23.4)	157 (23.6)	67 (22.9)		115 (23.0)	60 (24.0)	55 (22.0)	
	Mean (SD)	41.1 (13.8)	41.2 (13.8)	40.9 (13.7)	0.026	40.7 (14.2)	40.6 (14.4)	40.8 (14.1)	0.009
Sex	Female	561 (58.5)	400 (60.1)	161 (54.9)	0.104	276 (55.2)	135 (54.0)	141 (56.4)	0.048
	Male	398 (41.5)	266 (39.9)	132 (45.1)		224 (44.8)	115 (46.0)	109 (43.6)	
Marital status	Single	211 (22.0)	165 (24.8)	46 (15.7)	0.255 *	94 (18.8)	50 (20.0)	44 (17.6)	0.072
	Married	663 (69.1)	451 (67.7)	212 (72.4)		358 (71.6)	175 (70.0)	183 (73.2)	
	Separated	85 (8.9)	50 (7.5)	35 (11.9)		48 (9.6)	25 (10.0)	23 (9.2)	
Level of education	None	423 (44.1)	293 (44.0)	130 (44.4)	0.340*	228 (45.6)	115 (46.0)	113 (45.2)	0.138
	Primary	297 (31.0)	200 (30.0)	97 (33.1)		160 (32.0)	80 (32.0)	80 (32.0)	
	Secondary	121 (12.6)	72 (10.8)	49 (16.7)		71 (14.2)	31 (12.4)	40 (16.0)	
	Tertiary and above	118 (12.3)	101 (15.2)	17 (5.8)		41 (8.2)	24 (9.6)	17 (6.8)	
Has a source of income	No	604 (63.0)	396 (59.5)	208 (71.0)	0.244 *	341 (68.2)	169 (67.6)	172 (68.8)	0.026
	Yes	355 (37.0)	270 (40.5)	85 (29.0)		159 (31.8)	81 (32.4)	78 (31.2)	
Distance to health facility (km)	<5	105 (10.9)	65 (9.8)	40 (13.7)	0.333*	205 (41.0)	103 (41.2)	102 (40.8)	0.081
	5–10	316 (33.0)	194 (29.1)	122 (41.6)		60 (12.0)	33 (13.2)	27 (10.8)	
	>10	538 (56.1)	407 (61.1)	131 (44.7)		235 (47.0)	114 (45.6)	121 (48.4)	
Accessible health facility	No	787 (82.1)	533 (80.0)	254 (86.7)	0.180	416 (83.2)	203 (81.2)	213 (85.2)	0.107
	Yes	172 (17.9)	133 (20.0)	39 (13.3)		84 (16.8)	47 (18.8)	37 (14.8)	
Drinks alcohol	No	951 (99.2)	663 (99.5)	288 (98.3)	0.122	498 (99.6)	248 (99.2)	250 (100.0)	0.127
	Yes	8 (0.8)	3 (0.5)	5 (1.7)		2 (0.4)	2 (0.8)	0 (0.0)	
Smokes cigarettes	No	958 (99.9)	666 (100.0)	292 (99.7)	0.083	500 (100.0)	250 (100.0)	250 (100.0)	<0.001
	Yes	1 (0.1)	0 (0.0)	1 (0.3)					
Duration on ART (years)	<5	579 (60.4)	405 (60.8)	174 (59.4)	0.056	191 (38.2)	99 (39.6)	92 (36.8)	0.146
	5–9	362 (37.7)	250 (37.5)	112 (38.2)		301 (60.2)	145 (58.0)	156 (62.4)	
	>10	18 (1.9)	11 (1.7)	7 (2.4)		8 (1.6)	6 (2.4)	2 (0.8)	
Received Pre-ART counseling	No	13 (1.4)	10 (1.5)	3 (1.0)	0.043	7 (1.4)	4 (1.6)	3 (1.2)	0.034
	Yes	946 (98.6)	656 (98.5)	290 (99.0)		493 (98.6)	246 (98.4)	247 (98.8)	
Received pre-TPT counseling	No	48 (5.0)	33 (5.0)	15 (5.1)	0.008	27 (5.4)	16 (6.4)	11 (4.4)	0.089
	Yes	911 (95.0)	633 (95.0)	278 (94.9)		473 (94.6)	234 (93.6)	239 (95.6)	
Experienced TPT side effects	No	951 (99.2)	659 (98.9)	292 (99.7)	0.085	499 (99.8)	250 (100.0)	249 (99.6)	0.09
	Yes	8 (0.8)	7 (1.1)	1 (0.3)		1 (0.2)	0 (0.0)	1 (0.4)	

TPT: Tuberculosis preventive therapy; ART: Antiretroviral therapy; SMD: Standardized mean difference;

*denotes covariate imbalance with SMD>0.2.

Age, alcohol consumption, smoking, duration of ART, pre-ART, and pre- of tuberculosis preventive therapy counseling, and experience of tuberculosis preventive therapy-related side effects were the only characteristics that showed comparable distribution between participants with and without disclosed HIV status (Table 1). We achieved comparable distribution of all these variables in the propensity-score matched data between participants with and without disclosed HIV status (all, SMD<0.2).

**Fig 2:**
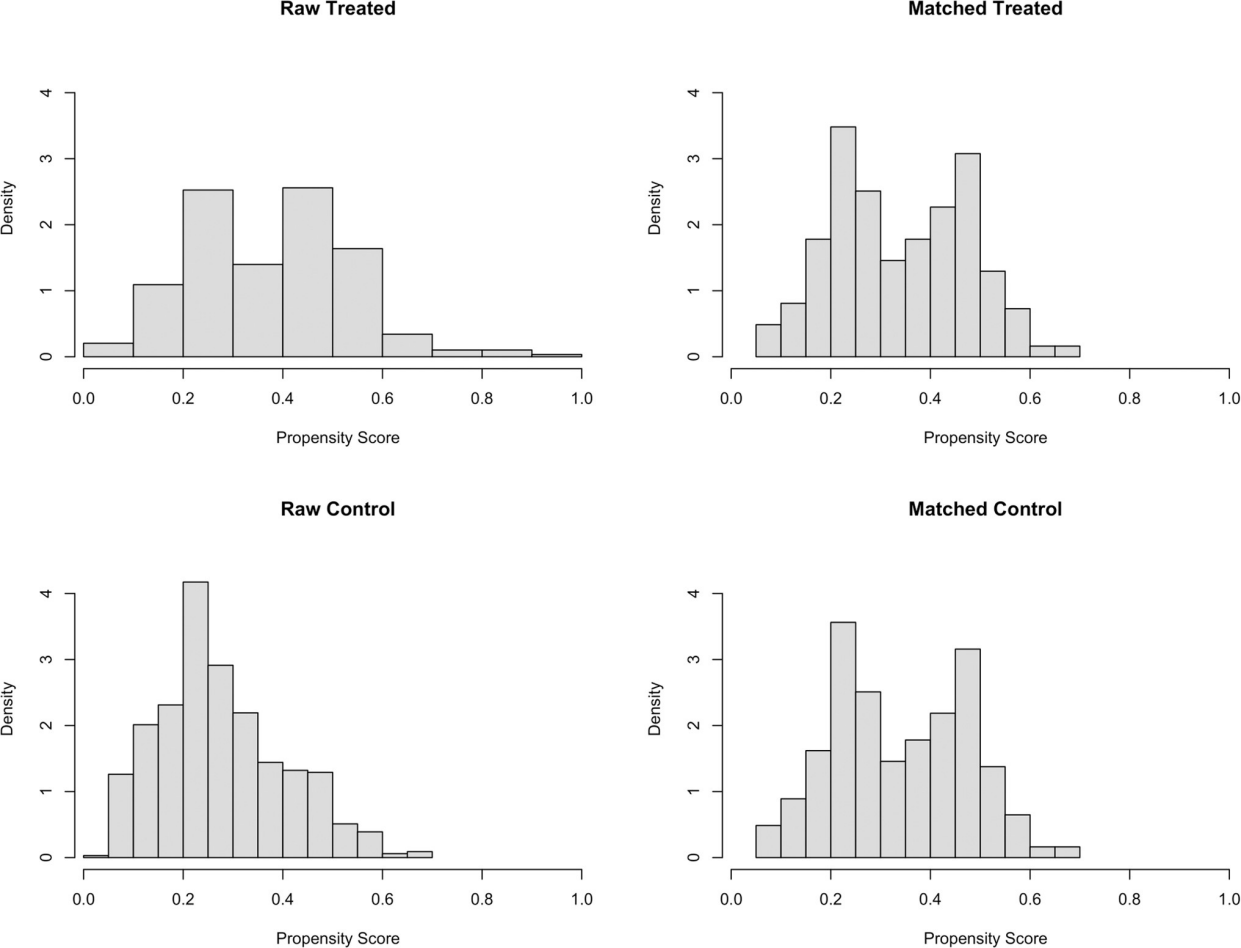
The histograms show the distribution of propensity scores before and after matching for the treated (disclosed HIV status) and untreated (undisclosed HIV status) groups. Raw treated: unmatched disclosed HIV status group; Raw control: Unmatched undisclosed HIV status group; Matched treated: matched disclosed HIV status group; Matched control: matched undisclosed HIV status group.

**Fig 3:**
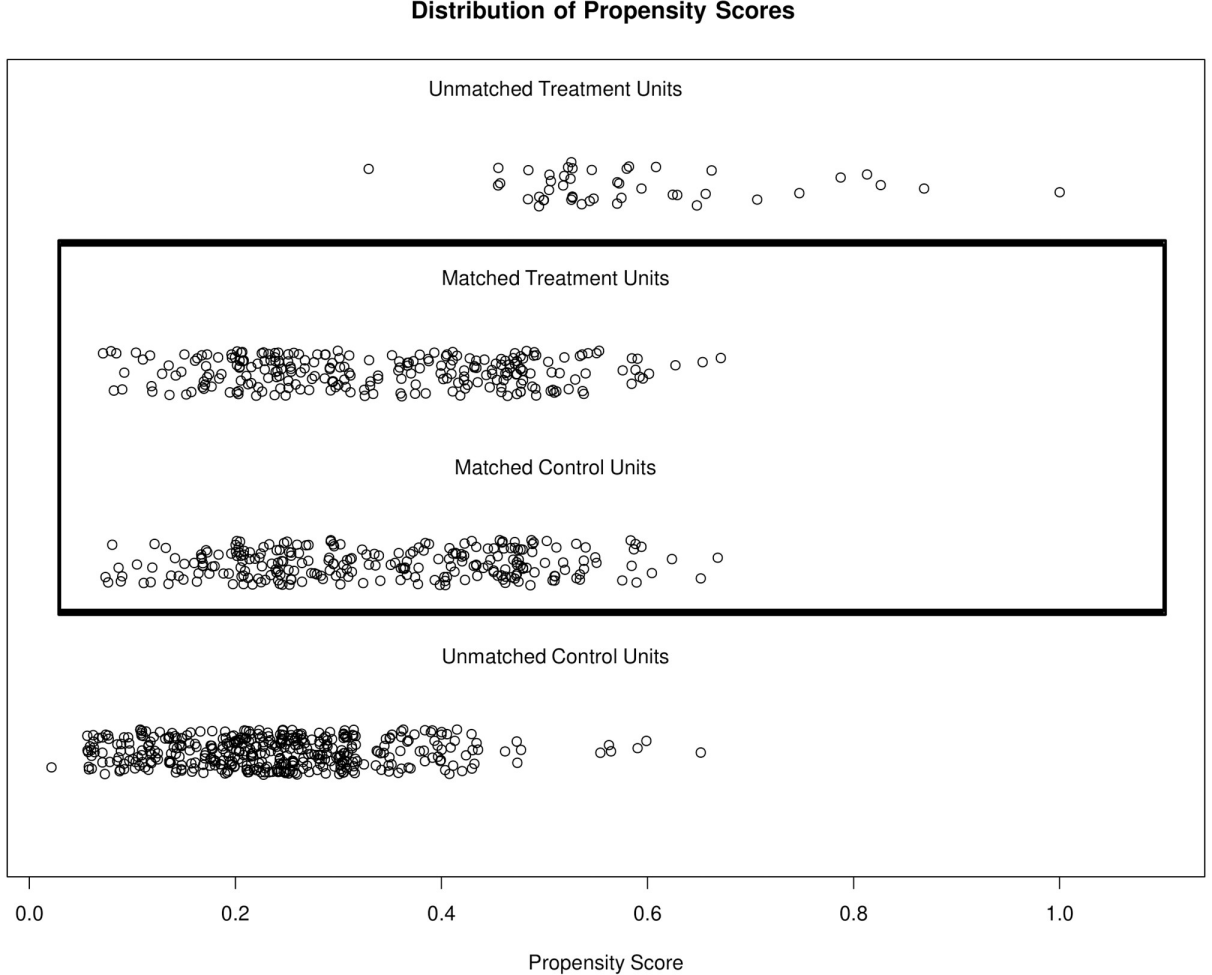
A jitter plot showing the distribution of the propensity scores before and after matching. Raw treated: unmatched disclosed HIV status group; Raw control: Unmatched undisclosed HIV status group; Matched treated: matched disclosed HIV status group; Matched control: matched undisclosed HIV status group.

Figs 2 and 3 show the distributional similarity of these covariates. Fig 2 (left-hand side) shows that in the unmatched data (raw treated versus raw control), the distribution of the propensity-scores across the disclosure of HIV status was not uniform suggesting an imbalance in covariates. However, after propensity-score matching (matched treated versus matched control), the right-hand side of Fig 2, the propensity-scores are similarly distributed across the disclosure of HIV status thus confirming the covariates are balanced. Fig 3 is a jitter plot showing the distribution of propensity scores before and after matching in both groups of participants. The middle section enclosed by a box shows that the treated (disclosed HIV status) and untreated (undisclosed HIV status) groups are similar after matching compared to before matching.

### Effect of disclosure of HIV status on adherence to clinic visits and patient representation

#### Unadjusted and adjusted outcome analysis

In the unmatched data (Table 2), the results show that adherence to clinic visits in the past 6 months was higher among those who disclosed compared to those with undisclosed HIV status: 129 (44.0%) versus 155 (23.3%), respectively (*p*<0.001). The regression analysis showed disclosure of HIV status was associated with an increased likelihood of adherence to clinic visits in the unadjusted (Unadjusted OR (uOR) = 2.59; 95% CI, 1.94–3.48) and adjusted analysis (Adjusted OR (aOR) = 2.04; 95% CI, 1.43–2.91). Patient representation was lower among participants with disclosed HIV status compared to those with undisclosed HIV status: 212 (72.4%) versus 534 (80.2%), respectively (*p* = 0.009). Disclosure of HIV status was associated with a 35% reduction in patient representation at unadjusted analysis (uOR = 0.65; 95% CI, 0.47–0.89) and by 45% at adjusted analysis (aOR = 0.55; 95% CI, 0.39–0.79).

**Table 2:** The effect of disclosure of HIV status on adherence to clinic visits and patient representation in the past 6 months.

		Original (unmatched) cohort				PSM matched cohort		
		HIV status disclosure		Unadjusted analysis	^+^Adjusted analysis	HIV status disclosure		PSM analysis
Outcome	Level	No	Yes	OR (95% CI)	aOR (95% CI)	No	Yes	OR (95% CI)
Adherence to clinic visits in the past 6 months	No	511 (76.7)	164 (56.0)	1	1	171 (68.7)	145 (58.2)	1
	Yes	155 (23.3)	129 (44.0)	2.59 *** (1.94, 3.48)	2.04 ** (1.43, 2.91)	78 (31.3)	104 (41.8)	1.63** (1.13, 2.36)
Patient representation in the past 6 months	No	132 (19.8)	81 (27.6)	1	1	47 (18.9)	71 (28.5)	1
	Yes	534 (80.2)	212 (72.4)	0.65 *** (0.47, 0.89)	0.55 *** (0.39, 0.79)	202 (81.1)	178 (71.5)	0.49** (0.32, 0.76)

Adjusted analysis included all matching covariates; 2) Statistical significance codes:

*** p<0.001,

** p< 0.01,

* p<0.05.

^+^: Adjusted for age, sex, marital status, level of education, source of income, distance from home to a health facility, alcohol consumption, smoking status, duration on ART, pre-ART counseling, pre-tuberculosis preventive therapy counseling, and tuberculosis preventive therapy-related side effects.

#### Propensity-score matched outcome analysis

In the propensity-score matched data (Table 2), the analysis showed that disclosure of HIV status is associated with higher odds of adherence to clinic visits (OR = 1.63; 95% CI, 1.13–2.36). Disclosure is associated with a more than 50% reduction in the odds of patient representation (OR = O.49; 95% CI, 0.32–0.76).

### Sensitivity analysis results

The sensitivity analysis results showed that in the presence of hidden bias and confounding, the unconfounded estimate was 0.3815 corresponding to a gamma value of 1. A gamma value of 2.2 was needed to achieve a statistically significant lower bound of 0.04, a point at which hidden bias is evident or where propensity-score matched analysis fails to remove confounding. Since a large increase in gamma value is needed to achieve statistical significance, this indicates that our results are robust to unobserved confounders and the matching approach.

## Discussion

We evaluated the effect of disclosure of HIV status on adherence to HIV clinic visits and patient representation. We found that disclosure of HIV status is associated with a higher likelihood of adherence to clinic visits and a lower likelihood of patient representation. Adherence to clinic visits is an important component of HIV clinical care because it enables HIV care providers to follow-up on the patient’s clinical progress, presents an opportunity for the patient to receive valuable information about their health from the attending healthcare provider, and is an indirect measure of the patient’s attitude towards health care. Adherence to clinic visits is important because previous studies have shown that it is associated with good drug adherence and clinical progress [33]. In other studies, missed appointments are associated with detectable viral load and poor immune recovery [34, 35]. Adherence to clinic visits is a proxy measure for adherence to ART [36, 37] because missed visits translate to a lack of medicines. In a recent Ghanaian study, Lokpo et al. (2020) observed statistically significant differences in viral suppression and adherence to clinic visits. The authors found that all the participants with detectable viral load had not adhered to clinic visits while those with undetectable viral load had adhered to clinic visits in the past 12 months [36]. These findings underscore the importance of adherence to clinic visits and low patient representation in achieving better outcomes of ART. Disclosure of HIV status might have mediated the association between HIV status disclosure and better outcomes of ART, namely undetectable viral load, immune recovery, and improvements in clinical status.

Although patient representation is one of the forms of clinic attendance, it has been associated with poor adherence to ART [38], and among hypertensive patients, the practice is neither associated with adherence to medications nor effective blood pressure control [39].

The lower patient representation among patients that have disclosed HIV status is a positive outcome of the disclosure. Individual patient attendance rather than patient representation is advantageous as it enables the uptake of ongoing psychosocial support, access to clinical reviews, laboratory and clinical monitoring for response to ART, and assessment of adherence to ART among others. Our findings of improved adherence to clinic visits and reduced patient representation could be explained by several factors. Disclosure of HIV status is encouraged among people living with HIV and they are supported to do so through ongoing psychosocial support. Therefore, it is likely that participants with disclosed HIV status have better social support, more control over their health, and are well prepared to face the challenges associated with disclosure of HIV status at both household and community levels.

Although our data show that disclosure of HIV status reduces patient representation, it should be noted that disclosure of HIV status is not mandatory in Uganda. Second, there is a possibility that some patients might have disclosed their HIV status to the representatives but we do not have sufficient data to support this claim. This should be a subject for further research.

### Methodological implications

We found similar results for the effect of disclosure of HIV status on adherence to clinic visits and patient representation for the two analytical approaches, although the measures of effect vary in magnitude. The measure of the effect for disclosure of HIV status on adherence to clinic visits in the unadjusted and adjusted analyses was attenuated in the PSM analysis. On the other hand, the effect of disclosure of HIV status was relatively lower in the unadjusted and adjusted analysis relative to the PSM analysis. These differences are likely attributable to confounding [40].

The results of the PSM analysis provide a more accurate measure of treatment effect compared to the unadjusted and adjusted logistic regression analyses.

### Study strengths and limitations

Our study has some important strengths. We used propensity-score matching and this approach enabled the estimation of unbiased effects of disclosure of HIV status since selection bias and confounding was removed. Our results are less likely to be biased by unmeasured confounders or the matching approach since sensitivity analysis showed robustness. Although our sample size reduced after the matching, the remaining sample size was relatively large to generate reliable conclusions since it met the minimum sample size for propensity-score matched analysis of 10(p+1), where p is the number of matching variables [41, 42]. However, there are limitations. We relied on secondary data which is prone to inaccuracies and missing entries. Our analysis did not include data on other confounders such as scheduling of clinic visits, participant’s functional status, and comorbidities during the review period among others. We did not examine the reasons for non-disclosure of HIV status as the data analyzed was secondary. Despite the limitations, to the best of our knowledge, this is the first study in Uganda to evaluate the effect of disclosure of HIV status on adherence to clinic visits and patient representation. Our findings strengthen the practice to encourage disclosure of HIV status in HIV programming.

## Conclusions and recommendations

Our data show that disclosure of HIV status improves adherence to clinic visits and reduces patient representation among people living with HIV in eastern Uganda. We recommend that people living with HIV should be supported through ongoing counselling to disclose their HIV status to improve adherence to clinic visits and reduce representation.

## Supporting information

S1 File Dataset. (DTA)

## References

[1] UNAIDS. Global HIV & AIDS statistics—2020 fact sheet. 2021 [cited 2021 Mar 16]. https://www.unaids.org/en/resources/fact-sheet

[2] World Health Organization. HIV/AIDS Geneva, Switzerland: World Health Organization.; 2020 [cited 2021 Feb 25]. https://www.afro.who.int/health-topics/hivaids

[3] Republic of Uganda. Consolidated guidelines for the prevention and treatment of HIV and aids in Uganda. Kampala, Uganda: Ministry of Health., 2020.

[4] Batte A, Katahoire AR, Chimoyi A, Ajambo S, Tibingana B, Banura C. Disclosure of HIV test results by women to their partners following antenatal HIV testing: a population-based cross-sectional survey among slum dwellers in Kampala Uganda. BMC Public Health. 2015;15(1):63. doi: 10.1186/s12889-015-1420-3 25637031 PMC4314734

[5] Ngonzi J, Mugyenyi G, Kivunike M, Mugisha J, Salongo W, Masembe S, et al. Frequency of HIV status disclosure, associated factors and outcomes among HIV positive pregnant women at Mbarara Regional Referral Hospital, southwestern Uganda. The Pan African medical journal. 2019;32. 10.11604/pamj.2019.32.200.12541 PMC6620078 31312312

[6] Naigino R, Makumbi F, Mukose A, Buregyeya E, Arinaitwe J, Musinguzi J, et al. HIV status disclosure and associated outcomes among pregnant women enrolled in antiretroviral therapy in Uganda: a mixed methods study. Reproductive health. 2017;14(1):107. doi: 10.1186/s12978-017-0367-5 28854944 PMC5577683

[7] Dessalegn NG, Hailemichael RG, Shewa-Amare A, Sawleshwarkar S, Lodebo B, Amberbir A, et al. HIV Disclosure: HIV-positive status disclosure to sexual partners among individuals receiving HIV care in Addis Ababa, Ethiopia. PloS one. 2019;14(2):e0211967. doi: 10.1371/journal.pone.0211967 30768642 PMC6415764

[8] Republic of Uganda. Consolidated guidelines for the prevention and treatment of HIV and aids in Uganda. Kampala, Uganda: Ministry of Health., 2018.

[9] Buma D, Bakari M, Fawzi W, Mugusi F. The Influence of HIV-Status Disclosure on Adherence, Immunological and Virological Outcomes among HIV-Infected Patients Started on Antiretroviral Therapy in Dar-es-Salaam, Tanzania. 2015.

[10] Bulali RE, Kibusi SM, Mpondo BC. Factors associated with hiv status disclosure and its effect on treatment adherence and quality of life among children 6–17 years on antiretroviral therapy in southern highlands zone, Tanzania: unmatched case control study. International journal of pediatrics. 2018;2018. doi: 10.1155/2018/8058291 30046314 PMC6038591

[11] Arrivé E, Dicko F, Amghar H, Aka AE, Dior H, Bouah B, et al. HIV status disclosure and retention in care in HIV-infected adolescents on antiretroviral therapy (ART) in West Africa. PloS one. 2012;7(3):e33690. doi: 10.1371/journal.pone.0033690 22457782 PMC3310064

[12] Akilimali PZ, Musumari PM, Kashala-Abotnes E, Kayembe PK, Lepira FB, Mutombo PB, et al. Disclosure of HIV status and its impact on the loss in the follow-up of HIV-infected patients on potent anti-retroviral therapy programs in a (post-) conflict setting: A retrospective cohort study from Goma, Democratic Republic of Congo. PloS one. 2017;12(2):e0171407. doi: 10.1371/journal.pone.0171407 28170410 PMC5295697

[13] Kimeu M, Burmen B, Audi B, Adega A, Owuor K, Arodi S, et al. The relationship between adherence to clinic appointments and year-one mortality for newly enrolled HIV infected patients at a regional referral hospital in Western Kenya, January 2011-December 2012. AIDS care. 2016;28(4):409–15. doi: 10.1080/09540121.2015.1109587 26572059 PMC4821878

[14] Alamo ST, Wagner GJ, Sunday P, Wanyenze RK, Ouma J, Kamya M, et al. Electronic medical records and same day patient tracing improves clinic efficiency and adherence to appointments in a community based HIV/AIDS care program, in Uganda. AIDS and behavior. 2012;16(2):368–74. doi: 10.1007/s10461-011-9996-9 21739285 PMC3872059

[15] Fong R, Cheng AC, Vujovic O, Hoy JF. Factors associated with virological failure in a cohort of combination antiretroviral therapy-treated patients managed at a tertiary referral centre. Sexual health. 2013;10(5):442–7. doi: 10.1071/SH13043 24119435

[16] Colubi MM, Pérez-Elías MJ, Elías L, Pumares M, Muriel A, Zamora AM, et al. Missing scheduled visits in the outpatient clinic as a marker of short-term admissions and death. HIV clinical trials. 2012;13(5):289–95. doi: 10.1310/hct1305-289 23134630

[17] Marbouh D, Khaleel I, Al Shanqiti K, Al Tamimi M, Simsekler MCE, Ellahham S, et al. Evaluating the Impact of Patient No-Shows on Service Quality. Risk management and healthcare policy. 2020;13:509. doi: 10.2147/RMHP.S232114 32581613 PMC7280239

[18] Uganda Bureau of Statistics. National population and housing census 2014-area specific profile series. Kampala, Uganda: 2017.

[19] Bonita R, Beaglehole R, Kjellström T. Basic epidemiology. Second ed: World Health Organization; 2006.

[20] White H, Sabarwal S. Quasi-Experimental Design and Methods: Methodological Briefs-Impact Evaluation No. 8. 2014.

[21] Okoli G, Sanders R, Myles P. Demystifying propensity scores. Oxford University Press; 2014. 10.1093/bja/aet290 PMC3854550 24318697

[22] Republic of Uganda. National adult HIV quality of care indicators. Kampala, Uganda: Ministry of Health, Uganda., 2014.

[23] R Core Team. R: A language and environment for statistical computing. R Foundation for Statistical Computing Vienna, Austria2018 [cited 2019]. https://www.R-project.org/.

[24] Ho DE, Imai K, King G, Stuart EA. MatchIt: nonparametric preprocessing for parametric causal inference. J Stat Softw. 2011;42(8):1–28.

[25] Yoshida K, Bohn J, Yoshida MK. Package ‘tableone’. R Foundation for Statistical Computing, Vienna, Austria (30 November 2016). 2020.

[26] Starks H, Garrido MM, editors. Observational & Quasi-experimental Research Methods. 8th annual Kathleen Foley palliative care retreat method workshop Google Scholar; 2004.

[27] Harris H, Horst SJ. A brief guide to decisions at each step of the propensity score matching process. Practical Assessment, Research, and Evaluation. 2016;21(1):4.

[28] Liu Y, Zumbo B, Gustafson P, Huang Y, Kroc E, Wu A. Investigating causal DIF via propensity score methods. Practical Assessment, Research, and Evaluation. 2016;21(1):13.

[29] Olmos A, Govindasamy P. Propensity Scores: A Practical Introduction Using R. Journal of MultiDisciplinary Evaluation. 2015;11(25):68–88.

[30] Randolph JJ, Falbe K. A step-by-step guide to propensity score matching in R. Practical Assessment, Research & Evaluation. 2014;19.

[31] Staffa SJ, Zurakowski D. Five steps to successfully implement and evaluate propensity score matching in clinical research studies. Anesthesia & Analgesia. 2018;127(4):1066–73. doi: 10.1213/ANE.0000000000002787 29324498

[32] Austin PC. Balance diagnostics for comparing the distribution of baseline covariates between treatment groups in propensity‐score matched samples. Statistics in medicine. 2009;28(25):3083–107. doi: 10.1002/sim.3697 19757444 PMC3472075

[33] Park WB, Choe PG, Kim SH, Jo JH, Bang JH, Kim HB, et al. One-year adherence to clinic visits after highly active antiretroviral therapy: a predictor of clinical progress in HIV patients. Journal of internal medicine. 2007;261(3):268–75. doi: 10.1111/j.1365-2796.2006.01762.x 17305649

[34] Rastegar DA, Fingerhood MI, Jasinski D. Highly active antiretroviral therapy outcomes in a primary care clinic. AIDS care. 2003;15(2):231–7. doi: 10.1080/0954012031000068371 12856344

[35] Lucas GM, Chaisson RE, Moore RD. Highly active antiretroviral therapy in a large urban clinic: risk factors for virologic failure and adverse drug reactions. Annals of internal medicine. 1999;131(2):81–7. doi: 10.7326/0003-4819-131-2-199907200-00002 10419445

[36] Lokpo SY, Ofori-Attah PJ, Ameke LS, Obirikorang C, Orish VN, Kpene GE, et al. Viral Suppression and Its Associated Factors in HIV Patients on Highly Active Antiretroviral Therapy (HAART): A Retrospective Study in the Ho Municipality, Ghana. AIDS Research and Treatment. 2020;2020.

[37] Shumba C, Atuhaire L, Imakit R, Atukunda R, Memiah P. Missed doses and missed appointments: adherence to ART among adult patients in Uganda. International Scholarly Research Notices. 2013;2013. doi: 10.1155/2013/270914 24052886 PMC3767323

[38] Kunutsor S, Walley J, Katabira E, Muchuro S, Balidawa H, Namagala E, et al. Clinic Attendance for Medication Refills and Medication Adherence amongst an Antiretroviral Treatment Cohort in Uganda: A Prospective Study. AIDS Res Treat. 2010;2010:872396. doi: 10.1155/2010/872396 21490907 PMC3065731

[39] Ogedegbe G, Schoenthaler A, Fernandez S. Appointment-keeping behavior is not related to medication adherence in hypertensive African Americans. Journal of general internal medicine. 2007;22(8):1176–9. doi: 10.1007/s11606-007-0244-y 17549574 PMC2305751

[40] Littnerova S, Jarkovsky J, Parenica J, Pavlik T, Spinar J, Dusek L. Why to use propensity score in observational studies? Case study based on data from the Czech clinical database AHEAD 2006–09. Cor et Vasa. 2013;55(4):e383–e90.

[41] Tumlinson SE, Sass DA, Cano SM. The search for causal inferences: using propensity scores post hoc to reduce estimation error with nonexperimental research. Journal of pediatric psychology. 2014;39(2):246–57. doi: 10.1093/jpepsy/jst143 24464252

[42] Mason C, Sabariego C, Thắng ĐM, Weber J. Can propensity score matching be applied to cross-sectional data to evaluate Community-Based Rehabilitation? Results of a survey implementing the WHO’s Community-Based Rehabilitation indicators in Vietnam. BMJ Open. 2019:9:e022544. doi: 10.1136/bmjopen-2018-022544 30782679 PMC6361336

